# Taurine as an Early-Phase Disease-Modifying Candidate for Alzheimer’s Disease

**DOI:** 10.3390/ijms27041871

**Published:** 2026-02-15

**Authors:** Muhammad Kamal Hossain, Hyung-Ryong Kim

**Affiliations:** 1 Organoids Laboratory, Department of Pharmacology, College of Dentistry, Jeonbuk National University, Jeonju 54896, Republic of Korea; kamalhossain@jbnu.ac.kr; 2 Non-Clinical Evaluation Center Biomedical Research Institute, Jeonbuk National University Hospital, Jeonju 54907, Republic of Korea

**Keywords:** Alzheimer’s disease, taurine, amyloid beta, ER stress, synaptic function, neuroprotective, multitarget regulator, translational potential

## Abstract

Alzheimer’s disease is driven by converging pathological processes, including amyloid-β accumulation, tau dysfunction, synaptic failure, and chronic neuroinflammation, which emerge decades before clinical onset. Growing evidence supports the concept that early, upstream neuroprotective interventions may meaningfully alter disease trajectory in both sporadic and familial AD. Taurine, an endogenously abundant and clinically safe neuromodulator, has re-emerged as a promising multi-target regulator of AD-relevant pathways. Accumulating mechanistic data indicate that taurine modulates Aβ aggregation, attenuates oxidative and endoplasmic reticulum stress, preserves mitochondrial homeostasis, suppresses neuroinflammatory signaling, and stabilizes synaptic function, positioning it as a promising upstream intervention strategy in AD. This review synthesizes current evidence supporting taurine’s pleiotropic neuroprotective actions and discusses its translational potential as an early-stage, low-risk intervention to delay or prevent AD progression.

## 1. Introduction

Alzheimer’s disease (AD) is a progressive neurodegenerative disorder and the leading cause of dementia, characterized clinically by memory impairment, cognitive decline, and behavioral changes. Pathologically, AD is defined by extracellular accumulation of amyloid-β (Aβ) peptides forming plaques, intracellular aggregation of hyperphosphorylated tau protein into neurofibrillary tangles, synaptic dysfunction, neuroinflammation, mitochondrial impairment, and widespread neuronal loss. These molecular and cellular abnormalities emerge decades before overt clinical symptoms and interact in a complex, self-amplifying cascade that ultimately disrupts neuronal networks and cognitive function [1]. The complexity of AD pathogenesis necessitates early, multi-target neuroprotective interventions rather than single-pathway approaches such as Aβ or tau targeting, which have yielded limited clinical benefit [2,3]. Although recent advances in anti-amyloid antibodies and biomarker-driven diagnostics represent important progress, most treatments are initiated after substantial neurodegeneration has already occurred, when lost neural circuits cannot be restored [4]. In contrast, neuroprotective strategies can act upstream to attenuate Aβ aggregation, stabilize mitochondrial and calcium (Ca^2+^) homeostasis, reduce oxidative and endoplasmic reticulum (ER) stress, and preserve synaptic integrity [5,6,7,8]—thereby slowing disease progression before irreversible damage occurs. Because AD pathology begins decades before clinical symptoms emerge [9], early-phase neuroprotection, particularly with multi-target agents such as taurine, represents the most rational and achievable therapeutic strategy for both sporadic AD (sAD) and familial AD (fAD).

Unlike many nutraceutical compounds or dietary amino acids, taurine is highly concentrated in the central nervous system, actively transported across the blood–brain barrier, and tightly regulated during neurodevelopment and adulthood. Taurine uniquely functions as an osmoregulator, neuromodulator, mitochondrial stabilizer, and Ca^2+^-buffering agent, rather than merely serving as a metabolic substrate. Moreover, its long-standing clinical use in cardiovascular and metabolic disorders, coupled with excellent safety and tolerability even at high doses, distinguishes taurine as an unusually translationally tractable candidate for neurodegenerative disease intervention [10]. Taurine, traditionally viewed as a metabolic and neuromodulatory molecule, is emerging as a candidate with multi-system neuroprotective properties, excellent safety, and supportive evidence across in vivo and human organoid systems [10,11,12,13]. Rather than acting through a single receptor or enzyme, taurine influences multiple pathways that converge on Aβ toxicity, cellular resilience, proteostasis, and network-level neuronal function. Taurine has gained renewed attention due to its neuroprotective, antioxidant, osmoregulatory, and calcium-buffering properties. Recent studies including investigations in 5XFAD mice and human cerebral organoids suggest that taurine may directly interact with Aβ, attenuate toxicity, and modulate downstream degenerative processes [12]. These findings collectively position taurine as a biologically attractive molecule for addressing early AD pathology.

While taurine has been widely studied in metabolic and cardiovascular contexts, its emerging role as a multi-target neuroprotective agent in AD remains fragmented across mechanistic domains. This review synthesizes molecular, cellular, and network-level evidence to position taurine as an upstream disease-modifying strategy, bridging preclinical insights with translational relevance, particularly in the context of early intervention, combinatorial therapy, and metabolic resilience.

## 2. Early Phase Neuroprotective vs. Curative Approach in AD Treatment

Therapeutic strategies for AD broadly fall into two conceptual categories: curative approaches, which aim to eliminate core pathological lesions such as Aβ plaques or tau aggregates, and neuroprotective approaches, which seek to preserve neuronal and synaptic function despite ongoing pathology. While these strategies are not mutually exclusive, their underlying assumptions, clinical feasibility, and therapeutic implications differ substantially.

AD drug development is predominantly centered on a curative paradigm, particularly targeting Aβ and tau pathology [14]. This focus is driven by strong genetic and pathological evidence implicating Aβ dysregulation as an initiating event in both familial and sporadic AD [15], as well as the availability of measurable biomarkers and targetable molecular species. Monoclonal antibodies directed against Aβ can robustly reduce plaque burden and have demonstrated modest slowing of cognitive decline in early-stage patients, validating target engagement and disease relevance [16,17]. This approach aligns well with regulatory frameworks that prioritize biomarker-based endpoints and mechanistic specificity. However, the curative paradigm faces intrinsic biological and practical limitations. By the time clinical symptoms emerge, extensive synaptic loss, circuit disintegration, and neuronal vulnerability are already established, raising doubts about whether removal of a single pathological species can meaningfully reverse functional deficits. Moreover, amyloid- and tau-targeted therapies have shown limited efficacy in later disease stages and are associated with safety concerns, high costs, and restricted patient eligibility [18]. These challenges underscore the difficulty of achieving true disease reversal in a multifactorial, network-level. For example, a critical limitation of the amyloid-focused approach is the temporal mismatch between pathological targeting and clinical intervention. By the time cognitive symptoms are evident, synaptic loss, circuit disorganization, mitochondrial dysfunction, and neuroinflammation are already well established and may progress independently of amyloid burden. Indeed, synapse loss and network dysfunction correlate far more strongly with cognitive decline than amyloid plaque burden, indicating that amyloid removal alone is insufficient once downstream neurodegenerative cascades such as mitochondrial dysfunction, oxidative stress, and neuroinflammation are established [19,20,21], making them biologically compelling intervention points. Neuroprotective strategies may therefore offer broader applicability across disease stages and etiological subtypes, including sAD and fAD. Importantly, such approaches aim to delay or attenuate functional deterioration rather than eradicate pathology, a goal that may be more achievable within clinically relevant time frames.

The promise of neuroprotection lies in its alignment with the emerging recognition of AD as a systems-level disorder involving interconnected molecular and cellular networks. Agents with pleiotropic actions may stabilize neural circuits, extend the window of cognitive function, and synergize with pathology-targeting therapies [22]. However, neuroprotective approaches also face limitations, including challenges in defining clear molecular endpoints, difficulties in regulatory approval due to less direct biomarker readouts, and the risk of symptomatic benefit without durable disease modification. While curative approaches remain the dominant focus of AD therapeutics due to their mechanistic clarity and regulatory compatibility, accumulating evidence suggests that neuroprotective strategies may offer a more realistic and broadly applicable path toward meaningful clinical benefit. A rational future direction is likely to integrate both paradigms, combining early pathological targeting with sustained neuroprotection to preserve neuronal function and delay cognitive decline across the AD pathology.

Early detection of AD increasingly relies on fluid, imaging, and functional biomarkers that reflect core pathological processes before irreversible neurodegeneration occurs. In cerebrospinal fluid (CSF) and plasma, decreased Aβ_42_ or Aβ_42/40_ ratio reflects cerebral amyloid deposition, while elevated phosphorylated tau (p-tau) and total tau (t-tau) indicate tau pathology and neuroaxonal injury. Imaging biomarkers include amyloid PET and tau PET for direct visualization of protein aggregates, and structural MRI for detecting regional brain atrophy, particularly in the hippocampus and medial temporal cortex. Functional biomarkers, such as FDG-PET hypometabolism and electrophysiological measures, further capture synaptic and network dysfunction [23,24,25,26,27].

Although direct clinical evidence linking taurine supplementation to changes in these biomarkers is currently limited, taurine’s pleiotropic effects, including regulation of intracellular Ca^2+^ homeostasis, stabilization of mitochondrial membrane potential (ΔΨm), and respiratory chain function, attenuation of microglial and astrocytic inflammatory responses, modulation of inhibitory neurotransmission, and protection against excitotoxicity and osmotic stress, suggest potential indirect modulation of biomarker-relevant pathways [10]. These mechanistic effects position taurine as a candidate molecule for influencing early pathological cascades upstream of measurable biomarker alterations, warranting systematic investigation in biomarker-driven clinical studies.

## 3. Mechanistic Rationale for Taurine in AD

Current pharmacological treatments for AD primarily include symptomatic therapies such as acetylcholinesterase inhibitors such as donepezil, rivastigmine, galantamine and the NMDA receptor antagonist memantine, which offer modest and temporary cognitive benefits without altering disease progression [28]. More recently, disease-modifying monoclonal antibodies targeting Aβ such as aducanumab, lecanemab, and donanemab have demonstrated biomarker-level reductions in amyloid burden and modest slowing of cognitive decline in selected patient populations [29]. However, these approaches are limited by modest clinical efficacy, high cost, restricted eligibility, infusion-related burdens, and risks such as amyloid-related imaging abnormalities (ARIA) [30]. These limitations highlight the urgent need for safe, accessible, and mechanism-based adjunctive or preventive strategies that target broader neurodegenerative pathways. In contrast to current AD therapeutics that primarily target single pathological axes, particularly amyloid deposition, taurine represents a pleiotropic small molecule with the capacity to modulate multiple convergent neurodegenerative mechanisms, including mitochondrial dysfunction, calcium dyshomeostasis, oxidative stress, neuroinflammation, and synaptic instability [31]. As a naturally occurring amino sulfonic acid with an established safety profile, taurine may serve as a complementary or preventive strategy that augments disease-modifying approaches by targeting upstream cellular resilience and network-level vulnerability rather than downstream protein aggregates alone.

**Figure 1:**
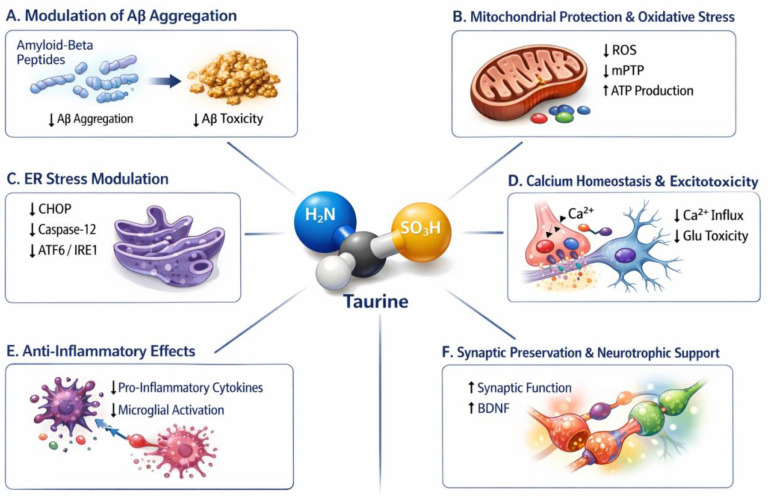
Neuroprotective mechanisms of taurine in AD. This schematic summarizes the multifaceted actions of taurine relevant to AD pathology. (**A**) Taurine modulates amyloid-β (Aβ) metabolism by reducing Aβ aggregation and associated synaptotoxicity. (**B**) Taurine confers mitochondrial protection, suppressing mPTP opening, decreasing reactive oxygen species (ROS) generation, and enhancing ATP production. (**C**) Taurine attenuates endoplasmic reticulum (ER) stress by downregulating CHOP, caspase-12, and ATF6/IRE1 signaling pathways. (**D**) Taurine contributes to Ca^2+^ homeostasis and limits network-level excitotoxicity by reducing Ca^2+^ influx and glutamatergic toxicity. (**E**) Taurine exhibits anti-inflammatory properties, decreasing pro-inflammatory cytokine production and microglial activation. (**F**) Taurine promotes synaptic preservation and neurotrophic support, enhancing synaptic function and upregulating brain-derived neurotrophic factor (BDNF). These convergent mechanisms highlight taurine as a pleiotropic neuromodulator with therapeutic potential in AD.

The therapeutic potential of taurine in AD has long been underestimated, largely because it has been perceived as a simple dietary nutrient rather than a pharmacologically relevant neuroactive molecule. This view was reinforced by the limited availability of molecular and systems-level analytical tools in earlier decades, the small size and heterogeneity of early clinical studies, and a field-wide bias toward highly specific, high-cost biologics. Taurine exerts multiple neuroprotective actions that together provide a strong mechanistic rationale for its potential benefit in AD. The coordinated regulation of synaptic function, redox balance, anti-inflammatory potential, mitochondrial integrity, ER stress, and Ca^2+^ signaling, taurine acts to attenuate Aβ-initiated neurotoxicity and supports neuronal survival in AD [31,32]. Figure 1 depicts the multifaceted neuroprotective role of taurine in AD. Moreover, recent advances in human organoid technologies now permit a more comprehensive assessment of taurine’s pleiotropic actions across Aβ and tau pathology [12]. As Alzheimer’s therapeutics increasingly shift toward interventions that modulate network-wide dysfunction rather than single pathogenic nodes, taurine aligns closely with this emerging paradigm, warranting renewed and rigorous investigation. An in-depth discussion of taurine’s multifaceted neuroprotective effects is provided in the following sections.

### 3.1. Modulation of Aβ Aggregation

The aggregation of Aβ peptides into soluble oligomers and fibrillar assemblies represents a critical upstream event in AD pathogenesis. A growing consensus now recognizes soluble Aβ oligomers, rather than mature plaques, as the primary neurotoxic species responsible for early synaptic failure and network dysfunction. Conversely, emerging evidence indicates that Aβ peptides are not universally pathological; rather, their effects are determined by aggregation state. While free Aβ species can support physiological synapse formation, aggregated Aβ42 assemblies are strongly neurotoxic and synaptotoxic. Therefore, instead of indiscriminately suppressing all Aβ, therapeutic strategies may aim to shift the balance from aggregated toward free Aβ species to preserve synaptic function while reducing toxicity [33]. Therefore, therapeutic strategies such as taurine that interfere with the initial stages of Aβ self-assembly offer greater early-phase disease-modifying potential than approaches aimed at late-stage plaque removal.

Accumulating evidence across human-relevant model systems positions taurine as a modulator of Aβ aggregation dynamics. In vitro, taurine has anti-amyloid beta activity by protecting mitochondrial function and neuronal cells through the activation of SIRT1 [34]. Moreover, data from the ThT assay also shows taurine reduces Aβ oligomerization and the formation of less dense, amorphous aggregates observed via TEM, indicating a capacity to alter peptide assembly kinetics [12]. In vivo studies in 5XFAD mice further demonstrate a reduction in amyloid plaque burden following taurine administration, suggesting sustained suppression of aggregation processes. For example, a study conducted by Jang et al. demonstrates that oral taurine administration at a relatively low dose (250 mg/kg/day for 10 days) ameliorates oligomeric Aβ-induced cognitive deficits in mice without altering locomotor activity. Taurine improved hippocampal-dependent memory performance in Y-maze and passive avoidance tests despite no reduction in Aβ plaque burden in prior work at higher doses. Importantly, surface plasmon resonance analyses revealed that taurine directly binds oligomeric Aβ, suggesting that its cognitive benefits are mediated through direct interaction with neurotoxic Aβ oligomers rather than global reduction in total Aβ levels [35]. However, Oh et al. demonstrate in vivo neuroprotection and network-level recovery, rather than binding specificity [36]. Together, previous work indicates that taurine not only confers neuroprotection against Aβ-induced toxicity but may also directly interact with oligomeric Aβ species. Evidence from behavioral, molecular, and imaging studies suggests that taurine ameliorates cognitive deficits through both direct binding to neurotoxic Aβ oligomers and functional preservation of neuronal networks. These convergent findings support taurine as a promising multimodal therapeutic candidate in AD, particularly for targeting oligomeric Aβ rather than simply reducing total amyloid burden. Notably, taurine also attenuates intracellular Aβ accumulation in patient-derived cerebral organoids [12], an experimental context that captures early AD-associated hallmarks. A recent study shows that taurine nanomicelles formulation effectively reduces the aggregation of Aβ peptides, particularly Aβ_1–42_ monomers, without exhibiting cytotoxicity in normal brain cells [37], indicating a promising therapeutic intervention against AD.

Furthermore, rather than acting as a high-affinity amyloid-binding agent, taurine appears to exert its effects by reshaping the physicochemical and cellular environment that governs Aβ misfolding. As a zwitterionic amino sulfonic acid with established roles in osmoregulation and ion homeostasis [38], taurine may stabilize local ionic conditions that otherwise favor β-sheet formation and oligomer nucleation [39]. Perturbations in calcium and sodium signaling are known to accelerate Aβ aggregation and exacerbate oligomer toxicity [7]; taurine-mediated buffering of these ionic microenvironments may therefore indirectly constrain aggregation-prone conformations. In parallel, transient interactions between taurine and aggregation-prone regions of the Aβ peptide may limit intermolecular β-sheet stacking, thereby suppressing the formation of toxic oligomeric intermediates. Notably, taurine has been shown to interact directly with Aβ assemblies at the molecular level. Molecular docking simulations indicate that taurine binds stably to multiple Aβ fragments, including the pentameric β-sheet fragment of Aβ_17–42_, primarily through hydrogen bonding and attractive charge interactions. Complementary molecular dynamics simulations further suggest that, although taurine forms a stable complex with the Aβ_17–42_ dimer, it can promote dissociation of the dimer interface over time [12]. These findings imply that taurine may hinder the formation and persistence of small, neurotoxic Aβ oligomers, thereby modulating Aβ aggregation toward less pathogenic states. This indirect mode of action distinguishes taurine from current antibody-based or small-molecule amyloid-targeting therapies. While monoclonal antibodies effectively reduce plaque burden, their limited efficacy in halting cognitive decline and their association with amyloid-related imaging abnormalities underscore the challenges of late-stage amyloid clearance. By contrast, taurine’s capacity to reduce early aggregation processes without forcibly removing deposited amyloid suggests a more physiologically aligned and potentially safer strategy, particularly in preclinical AD.

Current data support efficacy across in vitro systems, transgenic mouse models, and patient-derived organoids—combined with a long-standing safety profile—positions taurine as a compelling candidate for early, upstream intervention in AD, consistent with a shift toward preventative and disease-modifying therapeutic paradigms.

### 3.2. Mitochondrial Protection and Reduction in Oxidative Stress

Mitochondrial dysfunction and oxidative stress are among the earliest and most pervasive pathological features of AD, preceding overt amyloid plaque deposition and synaptic loss [40,41,42]. Accumulating evidence indicates that soluble Aβ species directly impair mitochondrial bioenergetics by disrupting electron transport chain (ETC) activity, depolarizing ΔΨm, and promoting excessive ROS generation [43]. Recent studies further implicate aberrant mitochondrial reverse electron transport (RET) as a major source of ROS production and NAD^+^/NADH imbalance in AD, with RET inhibition reducing amyloid burden, tau pathology, neuroinflammation, and cognitive deficits in AD mouse models and human iPSC-derived neurons [44]. Together, these alterations initiate a feed-forward cycle of oxidative damage, metabolic failure, and neuronal vulnerability that accelerates neurodegeneration in both sporadic and familial AD.

Given taurine’s established role in regulating mitochondrial function and redox homeostasis, it has emerged as a candidate neuroprotective agent capable of mitigating early mitochondrial stress in AD. In neuronal models exposed to Aβ_1–42_, taurine pretreatment significantly reduced mitochondrial membrane depolarization, inhibited opening of the mitochondrial permeability transition pore, restored ATP production, and attenuated ROS generation and Ca^2+^ overload, thereby improving neuronal survival. Notably, taurine also restored Aβ-suppressed SIRT1 expression, an effect abolished by SIRT1 knockdown, implicating SIRT1 signaling in taurine-mediated mitochondrial protection [34]. These findings indicate that taurine stabilizes mitochondrial function while limiting oxidative and Ca^2+^-mediated stress in Aβ-challenged neurons.

**Figure 2:**
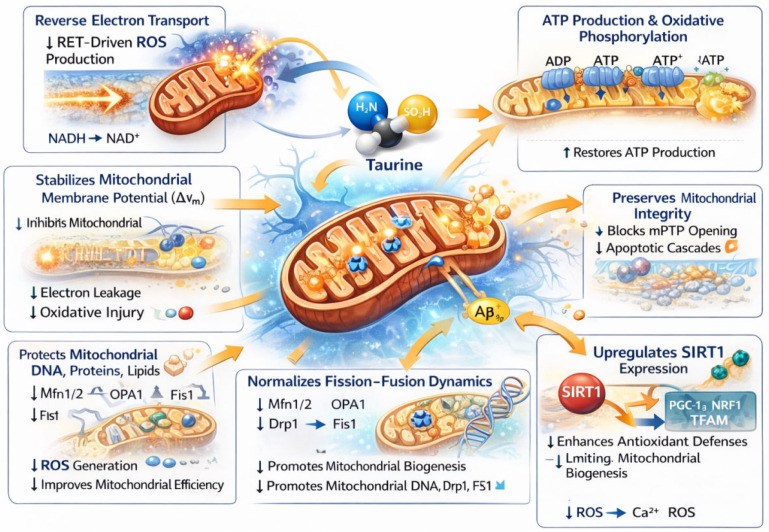
Taurine-mediated mitochondrial protection and attenuation of oxidative stress in AD. This schematic illustrates the major mitochondrial mechanisms through which taurine is proposed to exert neuroprotective effects in AD. Taurine enhances ATP production and oxidative phosphorylation, thereby restoring cellular energy metabolism and supporting neuronal function. It stabilizes mitochondrial membrane potential (ΔΨm), reducing electron leakage and oxidative injury. Taurine decreases reverse electron transport-driven reactive oxygen species (ROS) generation and protects mitochondrial DNA, proteins, and lipids from oxidative damage, ultimately improving mitochondrial efficiency. In addition, taurine preserves mitochondrial integrity by inhibiting mitochondrial permeability transition pore (mPTP) opening and suppressing downstream apoptotic cascades. It normalizes mitochondrial fission–fusion dynamics through regulation of Mfn1/2, OPA1, Drp1, and Fis1, promoting mitochondrial biogenesis and genomic maintenance. Taurine also upregulates SIRT1 expression, activating PGC-1α, NRF1, and TFAM, which enhances antioxidant defenses and supports mitochondrial biogenesis. These actions mitigate Aβ-induced mitochondrial dysfunction, decrease ROS and Ca^2+^ dysregulation, and contribute to improved neuronal survival in Alzheimer’s disease.

Beyond these AD-focused studies, taurine has been shown more broadly to stabilize mitochondrial membranes [45,46], preserve ETC efficiency, and maintain oxidative phosphorylation under conditions of metabolic stress. Limited to AD study, taurine has been widely reported to exhibit antioxidant properties and to safeguard mitochondrial function under conditions of oxidative stress [47,48,49]. However, the precise molecular mechanisms through which taurine confers mitochondrial protection are not yet fully elucidated. Although, taurine is not a classical free radical scavenger [50], it reduces ROS accumulation by improving mitochondrial efficiency and limiting electron leakage from the ETC. Taurine also modulates redox-sensitive signaling pathways and attenuates oxidative stress-induced damage to mitochondrial DNA, proteins, and lipids [51], processes closely linked to early neuronal dysfunction in AD. Importantly, recent studies suggest that taurine influences mitochondrial dynamics by regulating the balance between fission and fusion processes. Excessive mitochondrial fragmentation is increasingly recognized as a contributor to synaptic degeneration and neuronal loss in AD. Taurine-mediated normalization of fission–fusion dynamics may therefore preserve mitochondrial network integrity, enhance mitochondrial trafficking to synapses, and sustain local energy supply under AD pathological stress conditions. Consistent with our proposed mitochondrial mechanism, in non-AD mouse models, long-term taurine supplementation improved mitochondrial biogenesis and dynamics through modulation of PGC-1α, NRF1, TFAM, Mfn1/2, OPA1, Drp1, and Fis1, alongside enhanced cognitive performance and behavioral outcomes [52]. Although not conducted in AD models, these findings support a broader role for taurine in maintaining mitochondrial network integrity and bioenergetic resilience. Figure 2 illustrates the major mitochondrial mechanisms through which taurine is proposed to exert neuroprotective effects in AD.

Collectively, these data indicate that taurine acts upstream of irreversible neurodegenerative cascades by stabilizing mitochondrial function, reducing oxidative stress, and preserving bioenergetic homeostasis. While clinical efficacy in AD remains to be established, taurine’s endogenous nature, favorable safety profile, and multi-target mitochondrial actions position it as a rational candidate for early-stage disease-modifying strategies aimed at mitigating mitochondrial vulnerability in AD.

### 3.3. ER Stress Modulation

ER stress is an early and convergent pathological feature of AD, emerging before overt synaptic degeneration and neuronal loss [53,54]. Accumulation of misfolded proteins, including toxic Aβ species, disrupts ER proteostasis [55,56] and chronically activates the unfolded protein response (UPR) [57], which is initially protective but becomes maladaptive when sustained [58]. Experimental and postmortem studies show that abnormal APP and PSEN1 processing promote ER stress, PERK–eIF2α signaling, and CHOP-mediated apoptosis, contributing directly to synaptic vulnerability in both sporadic and familial AD [59,60,61].

Because dysregulated ER stress and maladaptive UPR contribute to AD pathogenesis, they represent promising targets for therapy. Naturally derived compounds such as taurine that can mitigate ER stress may therefore offer potential strategies to correct maladaptive UPR in AD. Taurine presents a mechanistically plausible modulator of ER stress by selectively dampening maladaptive UPR signaling while preserving homeostatic capacity [31,62,63]. Experimental evidence mechanistically indicates that taurine has been shown to suppress ER stress-induced apoptotic pathways relevant to neurodegeneration. In aged rats, isoflurane anesthesia triggered robust activation of ER stress sensors (GRP78, p-IRE1, cleaved ATF6, CHOP) and downstream apoptotic mediators, accompanied by cognitive deficits. Taurine pretreatment dose-dependently attenuated these ER stress responses and normalized apoptosis-related proteins, including Bcl-2/Bax ratio, cytochrome-c release, and caspase-3 activation. Importantly, taurine preserved spatial working memory performance in these animals by preventing hippocampal-dependent cognitive impairment [64]. These findings support ER stress modulation as a key mechanism through which taurine confers neuroprotection.

Moreover, taurine protects neurons in part through regulation of the ER stress response. Experimental studies in primary cortical neurons have shown that taurine suppresses hypoxia/reoxygenation-induced increases in caspase-12 and CHOP/GADD153, key mediators of ER stress-driven apoptosis. In addition, taurine downregulates cleaved ATF6, full-length ATF6, and phosphorylated IRE1, indicating inhibition of both the ATF6 and IRE1 branches of the unfolded protein response. These data support taurine as an ER stress modulator capable of limiting neuronal death under excitotoxic and hypoxic conditions relevant to AD [65]. Importantly, this modulatory effect contrasts with broad-spectrum UPR inhibitors, which often compromise adaptive stress responses and exacerbate neuronal dysfunction. Taurine therefore appears to recalibrate, rather than abrogate, ER stress signaling—an essential distinction for long-term neuroprotection. In addition, ER stress in AD is tightly coupled to mitochondrial dysfunction through disruption of mitochondria-associated ER membranes (MAMs), amplifying Ca^2+^ dysregulation, bioenergetic failure, and ROS production [7]. Taurine supports ER–mitochondria coupling by stabilizing intracellular Ca^2+^ handling and preserving ΔΨm [66], thereby constraining feed-forward proteotoxic stress. This coordinated regulation positions taurine as a systems-level modulator of proteostasis rather than a single-pathway intervention.

From a therapeutic perspective, ER stress modulation aligns with the emerging consensus that effective AD intervention (Table 1) must occur upstream of irreversible neurodegeneration. Collectively, taurine modulates ER stress by selectively dampening maladaptive UPR signaling while preserving adaptive proteostatic responses, thereby limiting ER stress-induced apoptosis and neuronal vulnerability. Its ability to regulate PERK, IRE1, and ATF6 pathways and stabilize ER–mitochondrial Ca^2+^ coupling positions taurine as a mechanistically grounded modulator of proteostasis relevant to early AD pathology.

### 3.4. Ca^2+^ Homeostasis and Network-Level Control of Excitotoxicity

Perturbations in neuronal Ca^2+^ homeostasis represent a unifying and early pathological axis in AD, linking Aβ toxicity, synaptic failure, network hyperexcitability, and neuronal vulnerability. Therefore, maintaining Ca^2+^ balance in neuronal cell could represents a promising therapeutic strategy for AD [7]. Converging evidence from genetic, imaging, and electrophysiological studies indicates that aberrant Ca^2+^ signaling precedes overt neurodegeneration and contributes to disease initiation rather than merely reflecting downstream damage. Excessive Ca^2+^ influx through NMDA receptors and voltage-gated Ca^2+^ channels, compounded by impaired ER and mitochondrial Ca^2+^ handling, drives excitotoxic stress and destabilizes synaptic networks in both sporadic and familial AD [68,69].

Within this framework, taurine emerges as a neuromodulatory regulator of Ca^2+^ dynamics with the capacity to act upstream of irreversible degenerative cascades. Unlike classical receptor-specific antagonists, taurine exerts homeostatic control over neuronal excitability by attenuating pathological Ca^2+^ elevations while sparing physiological signaling [70,71,72,73]. Experimental studies indicate that taurine limits Ca^2+^ influx through modulation of voltage-gated Ca^2+^ channels and dampens NMDA receptor-mediated excitotoxic currents, thereby constraining sustained intracellular Ca^2+^ accumulation induced by Aβ oligomers. Taurine also reduces or normalizes glutamate-induced increases in intracellular Ca^2+^, suppresses Ca^2+^ influx (but not efflux), and inhibits the reverse mode of the Na^+^/Ca^2+^ exchanger rather than acting as an NMDA receptor antagonist. These effects occur independently of taurine uptake into cells, indicating an extracellular membrane-mediated mechanism [74]. This mode of action is conceptually attractive, as excessive Ca^2+^ signaling amplifies tau pathology, oxidative stress, and synaptic destabilization. At the circuit level, taurine further contributes to Ca^2+^ stability by reinforcing inhibitory neurotransmission through GABAergic and glycinergic pathways [75,76,77]. Network hyperexcitability and excitation–inhibition imbalance are increasingly recognized as early and potentially targetable features of AD, detectable even in presymptomatic mutation carriers. By enhancing inhibitory tone, taurine may normalize aberrant neuronal firing patterns and reduce excitotoxic load, offering a mechanism to preserve circuit integrity during early disease stages. Electrophysiological studies further indicate that taurine modulates neuronal network activity through glycine receptor-dependent mechanisms. In immature neocortical slices, taurine selectively activated GABAergic interneurons via glycine receptors, leading to a marked increase in postsynaptic current frequency and action potential firing in pyramidal neurons. These effects were abolished by strychnine and gabazine but not by glutamatergic antagonists, demonstrating that taurine enhances network excitability through glycine receptor-driven activation of GABAergic circuits rather than via glutamatergic signaling [76]. This mechanism provides a biological basis for taurine-mediated regulation of early network synchronization and inhibitory tone, processes that are critically disrupted in AD. Beyond membrane-associated signaling, taurine influences intracellular Ca^2+^ buffering and organelle resilience [66]. Together, these findings suggest that taurine stabilizes ER- Ca^2+^ handling and protects mitochondria from Ca^2+^ overload, thereby sustaining bioenergetic capacity and limiting stress-induced apoptotic signaling.

In sum, taurine’s ability to stabilize Ca^2+^ homeostasis across molecular, cellular, and network levels provides a strong mechanistic rationale for its use as an early-stage neuroprotective strategy in AD. By superseding upstream of excitotoxic amplification and synaptic collapse, taurine is consistent with emerging disease-modifying approaches that emphasize maintenance of neuronal and circuit resilience rather than late-stage lesion removal. The interconnection among Ca^2+^ dysregulation, ER stress, and mitochondrial failure in AD further highlights taurine’s potential as a systems-level modulator rather than a single-pathway agent.

### 3.5. Anti-Inflammatory Effects

Chronic neuroinflammation is now recognized as a deteriorative driver of AD progression rather than a mere secondary response to amyloid and tau pathology. Sustained activation of microglia and astrocytes promotes synaptic dysfunction, exacerbates neuronal injury, and amplifies pathological feedback loops through excessive production of pro-inflammatory cytokines and reactive oxygen species [78,79,80]. Genetic and transcriptomic studies further implicate immune-related pathways in both sporadic and familial AD [81,82,83,84], revealing neuroinflammation as a therapeutic-targeting axis, particularly at early disease stages. Recent multi-omics network analyses have refined the mechanistic understanding of Alzheimer’s disease beyond isolated molecular targets. By integrating GWAS risk loci with the STRING interactome and cortical transcriptomes, an AD gene network was resolved into functionally coherent clusters encompassing complement activation, immune responses, lipid metabolism, endocytosis, RNA metabolism, proteostasis, and synaptic function. Notably, immune- and complement-related clusters were highly enriched in microglial transcripts, highlighting microglial-driven neuroinflammation as a central mechanistic hub. The identification of down-regulated cholinergic pathways and disrupted proteostasis further underscores convergent synaptic and protein-handling deficits [84]. These network-level findings support a mechanism-based therapeutic framework in which agents that modulate neuroinflammation, proteostasis, mitochondrial stress, Ca^2+^ homeostasis, and synaptic integrity may exert multidomain benefit. In this context, taurine is particularly relevant because it intersects several of these dysregulated pathways, including complement-linked inflammation, ER stress, oxidative injury, and synaptic preservation, suggesting that taurine acts not on a single target but on multiple nodes within the AD molecular network.

Taurine exerts a multifaceted anti-inflammatory profile that distinguishes it from conventional single-target anti-inflammatory agents. Mechanistically, taurine dampens microglial overactivation by modulating intracellular Ca^2+^ signaling and redox homeostasis, thereby shifting microglial phenotypes away from a pro-inflammatory state [11]. Experimental models also demonstrate that it restores acetylcholinesterase and acetylcholine transferase activities and inactivates microglia-dependent neuroinflammation [85]. In traumatic brain injury (TBI) rat model, taurine treatment significantly reduces the expression and release of key inflammatory mediators, including tumor necrosis factor-α (TNF-α), interleukin-1β (IL-1β), and interleukin-6 (IL-6) [86], all of which are strongly implicated in synaptic loss and cognitive decline in AD. A unique and biologically compelling aspect of taurine-mediated immunomodulation is the formation of taurine chloramine (TauCl). During inflammatory responses, activated microglia and infiltrating immune cells generate hypochlorous acid via myeloperoxidase activity. Taurine rapidly reacts with this cytotoxic oxidant to produce TauCl, a more stable compound that acts as a potent endogenous anti-inflammatory mediator. TauCl has been shown to suppress NF-κB signaling, inhibit inflammasome activation, and limit excessive cytokine production, thereby functioning as a negative feedback regulator of neuroinflammation [87,88]. This endogenous conversion mechanism suggests that taurine does not merely block inflammation but actively reprograms inflammatory signaling toward resolution. Importantly, taurine’s anti-inflammatory actions appear to operate upstream of irreversible neurodegenerative cascades. By constraining chronic immune activation, taurine may indirectly reduce amyloid-induced synaptotoxicity and tau-associated neurodegeneration, positioning it as a rational candidate for early-stage intervention. This upstream mode of action contrasts with late-stage anti-inflammatory strategies that have largely failed in clinical trials, likely due to intervention after extensive neuronal damage has already occurred. Large randomized trials of nonsteroidal anti-inflammatory drugs (NSAIDs) and COX-2 inhibitors including naproxen, celecoxib, and rofecoxib, showed no cognitive improvement and, in some cases, increased adverse events when administered after symptom onset [89]. Similarly, disease-modifying effects were not observed in administration of glucocorticoids or other broad anti-inflammatory agents [90]. These failures are widely attributed to intervention occurring after extensive synaptic and neuronal loss, when neuroinflammatory processes may reflect downstream responses rather than primary drivers of pathology. This underscores the necessity of targeting inflammatory and stress pathways at earlier, upstream stages of disease progression. Moreover, recent work has revealed that taurine modulates innate immune function through an immunometabolic mechanism. Taurine increases intracellular spermine levels in macrophages, which in turn suppresses JAK1/2–STAT1 signaling, thereby inhibiting M1 macrophage polarization and interleukin-1β (IL-1β) production. Through this pathway, taurine attenuates systemic inflammation and macrophage-driven tissue injury [91], providing a mechanistic basis for its anti-inflammatory and disease-modifying potential. Further, its favorable safety profile strengthens the case for repositioning taurine as a preventive or early-phase therapeutic strategy aimed at restoring immune homeostasis rather than bluntly suppressing inflammatory responses.

### 3.6. Synaptic Preservation and Neurotrophic Support

Synaptic dysfunction is an early and central event in AD, preceding overt neuronal loss and correlating more strongly with cognitive decline than amyloid plaque burden or tau aggregation. Soluble Aβ oligomers and hyperphosphorylated tau disrupt synaptic signaling by impairing glutamatergic transmission, destabilizing postsynaptic density architecture, and weakening activity-dependent synaptic plasticity [92,93,94,95]. Within this context, therapeutic strategies that preserve synaptic integrity and reinforce neurotrophic signaling during early disease stages represent a rational approach to disease modification.

Taurine, a conditionally essential amino sulfonic acid with established neuromodulatory functions, has emerged as a potential synaptoprotective agent in AD. Accumulating experimental evidence indicates that taurine enhances the expression and stability of key synaptic proteins, including postsynaptic density protein-95 (PSD-95) and the presynaptic vesicle protein synaptophysin. However, there is no clear evidence that taurine stabilizes these proteins post-translationally. These proteins are critical for maintaining synaptic architecture, neurotransmitter release, and receptor anchoring, all of which are compromised in AD. Previous research showed that, in developing mouse hippocampal neurons, taurine treatment increased protein levels of PSD-95 and synapsin-1 (another synaptic marker) as measured by Western blot, suggesting enhanced synaptogenesis. Similarly, in primary hippocampal neurons, taurine significantly increased density of PSD-95 puncta and synapsin-1 puncta, which is consistent with upregulated expression/localization at synapses [96]. Another study conducted by Brittany M et al. demonstrates that in cultured rat cortical neurons, 1 mM taurine significantly increased synaptophysin expression and puncta intensity, indicating enhanced presynaptic protein expression. However, in the same cortical neuron study, taurine did not significantly increase PSD-95 intensity or puncta number at the concentrations tested, although trends were reported [97]. This suggests that the effect on PSD-95 may be context-dependent (e.g., cell type, taurine concentration, developmental stage). Contextual evidence suggests that, by preserving both pre- and postsynaptic components, taurine may counteract the structural synapse loss induced by Aβ oligomers and inflammatory mediators.

Beyond structural preservation, taurine exerts functional benefits through modulation of neurotrophic signaling pathways. Notably, taurine has been shown to enhance brain-derived neurotrophic factor (BDNF) expression and activate downstream cyclic AMP response element-binding protein (CREB) signaling [98,99,100]. The BDNF–CREB axis is essential for synaptic plasticity, dendritic spine maintenance, and memory consolidation, and its impairment is a hallmark of early AD [101]. By restoring BDNF availability and CREB phosphorylation, taurine may reinstate activity-dependent gene transcription required for synaptic maintenance and adaptive plasticity [102]. These molecular effects translate into functional improvements at the circuit level. Earlier research also supports that in AD in vivo models, taurine supplementation has been associated with the restoration of long-term potentiation (LTP) [103,104,105], a cellular correlate of learning and memory that is profoundly disrupted by Aβ-mediated synaptic toxicity. The recovery of LTP suggests that taurine not only preserves synaptic structures but also normalizes synaptic efficacy and network connectivity. Although taurine can act as an agonist at extrasynaptic GABA_*A*_ receptors and modulate thalamic network excitability [106], taurine-mediated synaptic rescue in neurodegenerative contexts appears to be primarily driven by receptor-independent mechanisms, including mitochondrial protection, antioxidant activity, and regulation of Ca^2+^ homeostasis. This proof of concept supports the idea that taurine acts as a homeostatic modulator rather than a conventional excitatory or inhibitory agent.

## 4. Taurine-Mediated Neuroprotection: Evidence from Preclinical Study

Recent preclinical studies provide compelling evidence that taurine exerts multi-level neuroprotective effects across diverse models of AD and related neurodegenerative disorders. In senescence-accelerated SAMP8 mice, taurine administration significantly reduced activated microglia, Aβ burden, and phosphorylated tau levels while upregulating triggering receptor expressed on myeloid cells 2 (TREM2) [107], indicating attenuation of neuroinflammation and enhanced microglial-mediated clearance of pathological aggregates. In parallel, taurine treatment in sporadic dementia of Alzheimer’s type (SDAT) rat models improved spatial memory performance and reduced hippocampal neuroinflammatory markers, further supporting its anti-inflammatory and cognition-preserving effects [108].

Mechanistically, taurine has been shown to directly interact with toxic oligomeric Aβ species. Oral taurine supplementation (~250 mg/kg) bound soluble Aβ oligomers and reversed cognitive deficits in Aβ-infusion mouse models [35], suggesting that taurine may mitigate early synaptotoxic events that precede plaque deposition. In transgenic APP/PS1 mice, chronic taurine intake via drinking water restored learning and memory in Y-maze and passive avoidance paradigms and modestly reduced insoluble Aβ accumulation, indicating disease-modifying potential at both behavioral and pathological levels [109]. Similarly, long-term dietary taurine supplementation in 5xFAD mice mitigated AD-related pathology [12], highlighting taurine’s capacity to reprogram metabolic vulnerability pathways associated with neurodegeneration. Beyond AD models, taurine also confers neuroprotection in Parkinson’s disease paradigms, where it reduced astroglial activation, preserved dopaminergic neuron integrity, and improved motor outcomes in MPTP- and MPP^+^-treated rodents, underscoring its broad-spectrum neuroprotective properties [110]. In another study, dietary taurine supplementation restores potassium ion balance, enhances mitochondrial function, and reduces PTSD-like behaviors in rat models [111]. At the cellular level, taurine activates Nrf2-dependent antioxidant pathways, including induction of heme oxygenase-1 (HO-1), thereby suppressing glutamate- and hydrogen peroxide-induced oxidative stress and apoptotic signaling in neuronal cultures [112]. Complementary in vivo imaging studies further demonstrate that taurine modulates glutamatergic neurotransmission, as evidenced by altered glutamate receptor uptake on functional PET imaging in AD mouse models, suggesting normalization of excitotoxic circuitry [36].

## 5. Current Research Gaps and Future Directions

Despite growing interest in taurine as a pleiotropic neuroprotective agent in AD, its mechanistic foundations remain insufficiently resolved to support rational therapeutic deployment. A major unresolved issue concerns the molecular specificity of taurine–Aβ interactions. Existing studies do not adequately define whether taurine directly engages discrete Aβ conformers or preferentially suppresses the oligomeric species most tightly linked to synaptic toxicity. Without such resolution, it remains unclear whether reported reductions in aggregation reflect primary anti-amyloid activity or secondary consequences of broader improvements in proteostasis, mitochondrial resilience, or redox balance. Unravelling these possibilities is essential to avoid over-attribution of causality.

Similarly, while taurine’s capacity to preserve mitochondrial integrity and attenuate oxidative stress is consistently reported [42,49,87], the upstream molecular mechanisms remain largely inferential. It is not yet established whether taurine directly regulates mitochondrial fission–fusion dynamics, bioenergetic flux, or mitophagy, or whether these effects arise indirectly through modulation of Ca^2+^ signaling, antioxidant buffering, or metabolic coupling. Crucially, whether mitochondrial stabilization alone is sufficient to confer durable cognitive protection in human AD or merely delays inevitable network failure remains an open and underexplored question. Taurine’s influence on ER stress and UPR signaling presents an additional layer of complexity that demands more precise interrogation. Current evidence implicates PERK-associated pathways [61], yet the direct molecular interfaces through which taurine modulates these stress sensors are poorly defined. More importantly, the field lacks a clear framework for distinguishing adaptive UPR tuning from maladaptive suppression, particularly in the context of chronic neurodegeneration. Resolving whether taurine confers uniform ER stress resilience across neuronal populations or selectively stabilizes vulnerable circuits will be critical for defining its therapeutic boundaries. The same lack of mechanistic resolution extends to taurine’s role in Ca^2+^ homeostasis. Although taurine is frequently described as a stabilizer of neuronal excitability [73,74], the molecular specificity of its interactions with distinct Ca^2+^ channel subtypes in human neurons remains undefined. Moreover, whether these effects translate into sustained network stabilization and cognitive benefit in vivo has yet to be rigorously demonstrated. These uncertainties reinforce the likelihood that taurine’s efficacy is highly context- and stage-dependent, with maximal benefit confined to preclinical or early symptomatic phases of AD—a limitation that must be explicitly tested rather than assumed.

Moreover, taurine’s immunomodulatory effects, while conceptually attractive, also require a more critical appraisal. The extent to which taurine chloramine (TauCl) is generated within the human AD brain, particularly at disease-relevant concentrations and temporal windows, remains poorly characterized. Given the distinct heterogeneity of microglial states across disease stages, sex, and genetic background, taurine’s anti-inflammatory actions may not be universally beneficial and could conceivably blunt adaptive immune responses if deployed indiscriminately. Longitudinal, single-cell, and functional immune profiling will be essential to define when and where taurine-mediated immunomodulation is advantageous. Although taurine-induced enhancement of BDNF–CREB signaling and synaptic protein preservation has been reported [101,102], these observations remain mechanistically underdetermined. The upstream drivers of neurotrophic reinforcement, whether mitochondrial, redox-dependent, Ca^2+^-mediated, or inflammation-linked, have not been conclusively identified. Equally important, it remains unclear whether synaptoprotection can meaningfully counteract advanced-stage synapse loss or whether taurine’s value lies primarily in delaying the earliest phases of synaptic destabilization. Despite robust mechanistic and efficacy data from cellular and animal models, clinical evidence supporting taurine in AD or mild cognitive impairment remains limited. To date, few well-powered randomized controlled trials (RCT) have directly evaluated taurine supplementation in cognitively impaired populations, and existing studies often lack biomarker stratification, longitudinal follow-up, or disease-stage specificity. This scarcity of rigorous clinical data represents a major translational gap and underscores the need for biomarker-driven, placebo-controlled trials to determine whether taurine’s pleiotropic neuroprotective effects observed in preclinical systems translate into meaningful cognitive and disease-modifying outcomes in humans. Moreover, human evidence supporting taurine supplementation as a neuroprotective intervention in AD remains inconclusive. For example, a large prospective population-based cohort [113] reported no significant association between midlife dietary or circulating taurine levels and long-term risk of dementia, AD, or vascular dementia over a 25-year follow-up period, suggesting that habitual taurine intake alone may not confer measurable protection at the population level. Consistent with these findings, meta-analyses of RCTs, largely conducted in non-neurodegenerative contexts, indicate that taurine supplementation across a broad dose range (0.2–6 g/day) does not reliably improve cognitive performance in healthy individuals or populations at increased metabolic or vascular risk [114,115]; however, these studies were frequently underpowered and employed heterogeneous outcome measures.

Clinical trials registered (NCT03410173, NCT06613542) to date evaluating taurine supplementation in aging or metabolic disorders with secondary cognitive endpoints remain exploratory, and none have yet demonstrated disease-modifying or clinically meaningful cognitive benefits in neurodegenerative populations. Comprehensive supplement reviews similarly conclude that available human data provide limited or inconsistent support for taurine’s direct neuroprotective efficacy, highlighting small sample sizes, short intervention durations, and lack of disease-specific biomarkers as major limitations. Notably, mechanistic human studies report reduced expression of taurine transporters in cells derived from AD patients [116], suggesting altered taurine homeostasis in disease states; however, causal links between taurine signaling dysfunction and neurodegeneration remain unproven. Collectively, these findings indicate that while taurine exhibits robust neuroprotective effects in preclinical models, its translational efficacy in human neurodegenerative disease has yet to be established, underscoring the need for adequately powered, biomarker-driven clinical trials in well-characterized patient populations.

Together, these gaps argue against viewing taurine as a broadly effective, stage-agnostic intervention. Instead, they highlight the need to reposition taurine within a precision neuroprotection framework—one that explicitly defines molecular targets, temporal windows, and network contexts of efficacy. Addressing these unresolved questions will determine whether taurine represents a genuinely transformative strategy for early AD.

## 6. Conclusions

This review advances the concept that effective AD modification requires early, upstream, and multi-target intervention. By simultaneously modulating oxidative stress, synaptic integrity, mitochondrial function, neuroinflammation, and proteostasis pathways, taurine emerges as a uniquely positioned neuromodulator capable of preserving neuronal network resilience. These properties distinguish taurine from traditional single-target strategies and support its evaluation as a preventive and disease-modifying therapy. However, its therapeutic efficacy is likely to be strongly dependent on disease stage, network context, and the timing of intervention. In early or prodromal stages, taurine’s antioxidant, mitochondrial-stabilizing, and calcium-buffering effects may preserve synaptic integrity and delay network destabilization, whereas in advanced disease, its impact may be more limited due to irreversible neuronal loss and circuit disintegration. In addition, taurine is well tolerated in humans at supplemental doses commonly used in research and practice, with regulatory authorities in several countries designating it as “generally recognized as safe” (GRAS) when used within recommended ranges. While clinical validation is required, taurine’s safety, translational potential, and multi-target mechanisms make it an attractive candidate for early intervention strategies in AD.

## Figures and Tables

**Table 1:** Possible key mechanisms of taurine in AD through ER-stress modulation.

Mechanistic Category	Possible Actions of Taurine	Proposed Functional Consequences in AD	Ref.
**Selective modulation of the unfolded protein response (UPR)**	Dampens maladaptive, prolonged UPR activation Preserves adaptive/homeostatic UPR signaling Prevents shift from protective to pro-apoptotic ER stress	Restores ER proteostasis and limits transition to neuronal apoptosis	[62,65]
**Inhibition of PERK–eIF2α–CHOP-mediated apoptosis**	Limits sustained PERK activation Reduces eIF2α phosphorylation and translational repression Suppresses CHOP expression and CHOP-driven apoptosis	Prevents synaptic vulnerability and neuronal loss	[67]
**Suppression of ER stress sensors and downstream UPR pathways**	Downregulates GRP78/BiP, p-IRE1, cleaved/full-length ATF6 Inhibits IRE1 and ATF6 branches of UPR	Reduces maladaptive ER-stress signaling cascades	[65]
**Reduction in ER stress-induced caspase activation**	Decreases caspase-12 activation Attenuates caspase-3 activation	Limits ER-stress-driven neuronal apoptosis	[64]
**Stabilization of Bcl-2/Bax ratio and mitochondrial integrity**	Shifts Bcl-2/Bax toward cell survival Prevents cytochrome-c release	Protects against mitochondria-mediated apoptosis linked to ER stress	[64]
**Attenuation of toxic Aβ-induced ER stress**	Interferes with ER stress triggered by misfolded Aβ oligomers and “toxic-turn” Aβ	Mitigates early ER proteostasis disruption in AD pathology	[59,64]
**Recalibration of ER–mitochondria crosstalk (MAM regulation)**	Preserves MAM function Stabilizes intracellular Ca^2+^ handling Maintains ΔΨm Limits amplification of Ca^2+^ dysregulation, oxidative stress, bioenergetic failure	Constrains feed-forward ER–mitochondrial injury loops	[66]
**Protection under hypoxic and excitotoxic stress conditions**	Suppresses CHOP and caspase-12 under hypoxia/reoxygenation	Enhances neuronal survival under stress relevant to AD	[65]
**Systems-level proteostasis modulation**	Rebalances ER-stress signaling rather than fully inhibiting UPR Avoids impairment of adaptive stress responses	Acts as network-level proteostasis modulator	[66]

## Data Availability

No new data were created or analyzed in this study. Data sharing is not applicable to this article.
